# Melatonin Application Modifies Antioxidant Defense and Induces Endoreplication in Maize Seeds Exposed to Chilling Stress

**DOI:** 10.3390/ijms22168628

**Published:** 2021-08-11

**Authors:** Izabela Kołodziejczyk, Andrzej Kaźmierczak, Małgorzata M. Posmyk

**Affiliations:** 1 Department of Plant Ecophisiology, Faculty of Biology and Environmental Protection, University of Lodz, 90237 Lodz, Poland; malgorzata.posmyk@biol.uni.lodz.pl; 2 Department of Cytophysiology, Faculty of Biology and Environmental Protection, University of Lodz, 90237 Lodz, Poland; andrzej.kazmierczak@biol.uni.lodz.pl

**Keywords:** antioxidant enzymes, biostimulation, chilling stress, corn seeds, endoreplication, melatonin, seed priming, *Zea mays*

## Abstract

The aim of the study was to demonstrate the biostimulating effect of exogenous melatonin (MEL) applied to seeds via hydroconditioning. It was indicated that only well-chosen application technique and MEL dose guarantees success concerning seed germination and young seedlings growth under stress conditions. For maize seed, 50 μM of MEL appeared to be the optimal dose. It improved seed germination and embryonic axes growth especially during chilling stress (5 °C/14 days) and during regeneration after its subsided. Unfortunately, MEL overdosing lowered IAA level in dry seeds and could disrupt the ROS-dependent signal transduction pathways. Very effective antioxidant MEL action was confirmed by low level of protein oxidative damage and smaller quantity of lipid oxidation products in embryonic axes isolated from seeds pre-treated with MEL and then exposed to cold. The stimulatory effects of MEL on antioxidant enzymes: SOD, APX and GSH-PX and on GST-a detoxifying enzyme, was also demonstrated. It was indicated for the first time, that MEL induced defence strategies against stress at the cytological level, as appearing endoreplication in embryonic axes cells even in the seeds germinating under optimal conditions (preventive action), but very intensively in those germinating under chilling stress conditions (intervention action), and after stress removal, to improve regeneration.

## 1. Introduction

The majority of plant cultivation in spite of various agrotechnical procedures is maintained in suboptimal conditions for plants, but also the changing climate exposes them to new stresses. Unfortunately, excessive agriculture chemicalization, application of fertilizers and synthetic plant protection products, are unfavorable for the environment. Hence exists the necessity of searching for natural (biodegradable) factors, that through induction of internal defense strategies, might help plants overcome or adapt to stresses [1,2].

One of the most important cultivated plants in the world is corn (*Zea mays* L.). The plant is an important part of human diet and, furthermore, in the face of the prevalence of food allergies, the absence of gluten in corn grains increases their value on the cereal market [3,4]. Moreover, green parts of corn are fodder for farm animals, and biodegradable, fully ecological materials are produced from those plant fibers. Because maize comes from South America, the climate requirements for its growth are not entirely achieved in temperate climate zone, even for adapted varieties. Particularly temperature conditions for germination are suboptimal and limiting [5], therefore every low-cost improvement of sprouting might bring a measurable increase in harvests and economic benefits.

The quality of seed material is a primary and basic criterion determining good yields. Thus, finding effective techniques to improve sowing material seems to be a crucial point to resist potential stressors [6,7]. Conditioning is a highly effective method for improvement of seed vigor [8]. However, to succeed, the water application method must be properly adjusted to a type (species) of seeds. Selection of the method should involve physiology, biochemistry and anatomy of seeds, the value of initial seed moisture and experimentally determined final value. The final moisture value is the one that initiates the germination process but does not allow an embryo to grow and promote secondary desiccation-the necessary step in any type of conditioning [9,10,11,12]. This technique can be combined with other supporting factors such as aeration, light-irradiation, and temperature-stratification. What is most important, various soluble bioactive substances (growth regulators, fungicides, biostymulators) can be applied to the seeds with water [2,13,14].

Many scientists, as well as breeders, consider that the development of new natural biostimulators is the most promising method for ecological crops production favoring healthy food and protection of the environment. Our research experience suggests that melatonin (MEL) has a significant phytobiostimulation potential, however, the effect of its, usually positive activity, is resultant of many factors [2,15]. The research on the MEL role in plants has been significantly intensified recently [16,17]. Scientists are interested both in (1) the role, regulation and endogenous phytomelatonin synthesis [17,18,19,20], and (2) effects of exogenous MEL application in plants, most often throughout supplementation of substrates, or spraying [21,22,23,24]. The presented work follows the second trend, yet in our research, MEL is applied to seeds via conditioning.

MEL antioxidative activity is well documented [25,26,27,28]. It detoxifies a variety of free radicals and reactive oxygen species (ROS) including hydroxyl radical (OH*), peroxynitrite anion (ONOO^−^), singlet oxygen (^1^O_2_*) and nitric oxide (NO) [29]. One of the most appealing properties of this molecule, which distinguishes it from most antioxidants, is the fact that its metabolites also have the ability to scavenge ROS and reactive nitrogen species (RNS) [30,31]. Melatonin generates a free radical scavenging cascade, ensuring continuous cell defense, which makes this molecule, even at low concentrations, highly effective in protecting organisms from oxidative stress [32,33]. There are also scientific reports suggesting the MEL influence on antioxidant enzymes activities [25,26,27,34] and their genes expression [35,36,37]—thereby on multidirectional effect on redox status in plant tissues. Direct and indirect antioxidant properties of MEL are particularly important in the face of environmental stresses. Various stressors inhibit plant growth by different mechanisms, however all of them cause increase in ROS concentration. Over-production of these reactive compounds, unfollowed by their neutralization, disturbs redox homeostasis and induces oxidative stress, which is a well-known secondary effect of all biotic and abiotic stresses. In addition to the fact that antioxidant activity is very desirable in stressed plant cells, it is worth mentioning, that ROS can also be formed naturally as products of biochemical reactions involved in various physiological cycles (e.g., water-water cycle, cell-wall biosynthesis and detoxification processes), play important role in signaling pathways in plants. Thus, they should not be completely eliminated but successfully restrained.

Oxidative stress blockade has a protective effect on biological membranes [38]. Owing to this property, MEL indirectly delays plant aging and limits the damages in chloroplasts and mitochondria [39,40,41].

The purpose of the presented work is to document the positive effects of MEL as a biostimulator supplemented during corn seed hydropriming, and study how exogenous melatonin can affect redox status of cells and modify antioxidant enzyme activity in embryonic axes isolated from corn seeds pretreated with MEL. Comparison was made between differently primed seeds variants: hydroconditioned with water (H), hydroconditioned with melatonin: 50 and 500 µM water solutions (HMel 50, HMel 500 respectively) and control—untreated ones (NT). There were three experimental procedures of germination for each seed variant: (i) optimal conditions at 25 °C (C), (ii) chilling stress at 5 °C (S), (iii) chilling stress with subsequent recovery at 25 °C (R).

Furthermore, our previous proteomic analyzes [14,42] have shown significant quantitative and qualitative differences between proteomes from embryos treated with MEL, untreated and water hydroconditioned. The results of these tests formed the grounds for the assumption, that enlarging the protein pool in MEL treated seed embryos can be a result of DNA amount multiplication. Therefore, in the presented work we investigate the correlation between the exogenous MEL application and the polyploidization in plants. We dealt with the issue of multiple replications of genetic material—endocycles.

We are the first to check whether MEL actually triggers the switchover of cell cycles to endocycles in maize. Based on densitometric measurements of the embryonic basal zones, after reference of fluorescence intensity of the genetic material in individual nuclei to the basic amount of DNA, histograms of genetic material distribution were created and a percentage of polyploid nuclei were obtained.

## 2. Results

### 2.1. Hydropriming Condition Determination

There are two ways to control the moisture of the seeds during imbibition: (1) by limiting the quantity of water added directly to the seeds or (2) by controlling the time of seed soaking.

**Figure 1:**
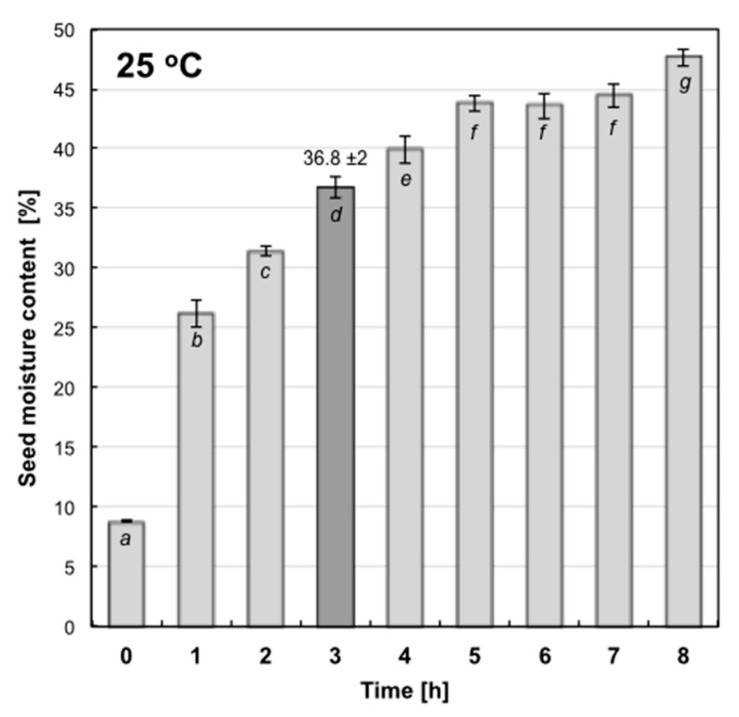
Kinetics of maize seed imbibition with distilled water at 25 °C. Vertical bars represent ±SEM (Samples of 60–100 seeds, *n* = 6). MC%—One-way ANOVA results: Time _(0–8h)_ F_(8;49)_ = 301.2, *p* < 0.000001.

In the former case, attention should be paid to homogeneous access of seeds to water amount given, calculated for their specific portion (weight) [43]. A limited amount of seeds (small portions) could be used in this technique and hydropriming should be carried out in rotating containers. Unfortunately, angular seeds, as dry corn, can be injured during rotations-as was observed in our preliminary experiments. In the latter case, when an unlimited quantity of water is used, the time of soaking to obtain experimentally established final seed moisture content should be precisely determined. In this case kinetics of imbibition at optimal temperature 25 °C was determined (Figure 1). It was shown that 3 h of corn seed soaking at 25 °C was sufficient to obtain 36.77% ± 2 of moisture content, thus hydropriming was done as follows: portions of 200 g of corn seeds were placed in plastic containers with 200 mL of oxygenated, distilled water or MEL water solutions (depending on the experimental variant) and incubated for 3 h at 25 °C. Then, the seeds were air-dried at room temperature for the subsequent 3 days (to let them return to the initial water content) and used in tests.

### 2.2. MEL and IAA Content in Seeds

HPLC-EC analyzes of NT, H, HMel 50 and HMel 500 seed extracts confirmed the efficacy of hydropriming supplemented with melatonin. The content of MEL increased 30 times in HMel 50 seeds and 600 times in HMel 500 ones compared to the untreated seeds. Hydropriming with water did not affect the amount of endogenous MEL—it remained at a level similar to that observed in NT seeds (Table 1).

Hydropriming with water provoked a statistically significant increase in the level of IAA in the seeds, while MEL applied during such treatment caused the inverse effect, i.e., the decrease in IAA content proportional to the concentration of MEL used during hydropriming (Table 1).

### 2.3. Seed Germination

**Figure 2:**
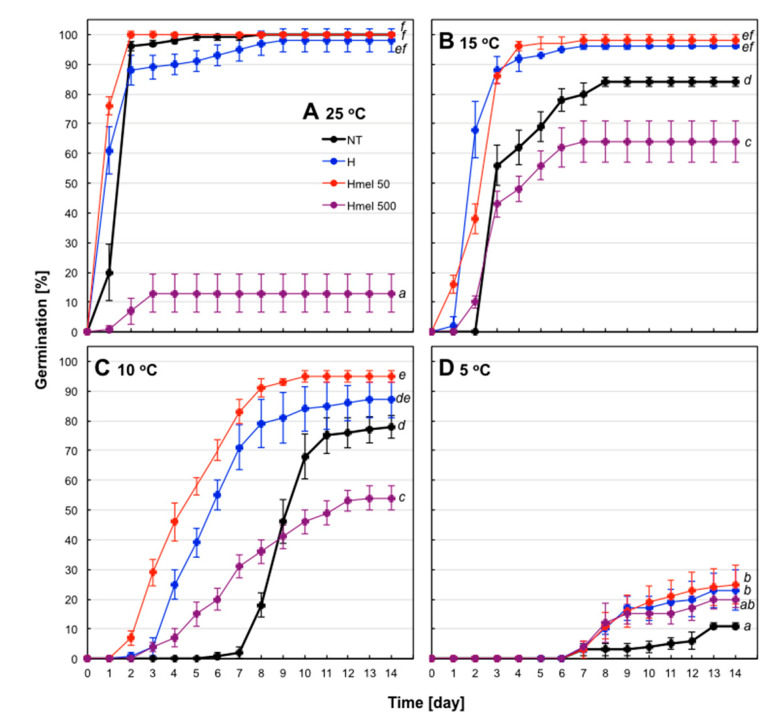
Effect of different maize seed hydropriming treatments on seed germination at optimal, 25 °C (**A**) and chilling: 15 (**B**), 10 (**C**) and 5 °C (**D**) temperatures. The seeds were hydroprimed with water (H—blue line) or with MEL water solution at concentrations 50 µM (HMel 50—red line) and 500 µM (HMel 500—purple line). Non-treated seeds (NT—black line) were not primed. Vertical bars represent ±SEM (Samples of 100 seeds; *n* = 4). GERMINATION 14d—ANOVA results: Seed Variant _(NT,H,HMel50,HMel500)_ F_(3;48)_ = 103.5, *p* < 0.000001; Temperature _(25,15,10,5)_ F_(3;48)_ = 266.6, *p* < 0.000001; interaction Seed Variant x Temperature F_(9;48)_ = 24.4, *p* < 0.000001.

Maize seeds used in the experiments were of good quality–in optimal conditions (25 °C) they achieved 96% of germination on the second day and 100% on the fifth day of the experiment (Figure 2A). Therefore, under these conditions, positive effects of pre-sowing treatments cannot be seen, as all variants of the seeds germinated well, the exception was HMel 500, with the surprising, only 13% germination (Figure 2A).

Lowering the temperature to 15 °C resulted in a slowdown of germination rate, the lag phase prolonged to 2 days and germination decreased to 84% in the control seeds—NT (Figure 2B). Germinability of H and HMel 50 seeds decreased very slightly—to 96% and 98%, respectively. Interestingly, at the suboptimal temperature, HMel 500 seeds began to germinate better than at optimal temperature, reaching a maximum of 64% on day 7 (Figure 2B).

Further lowering the temperature to 10 °C prolonged the lag phase to 6-7 days in NT seeds and reduced their germination to 77% (Figure 2C). In contrast, the conditioned seeds started to germinate on the 3rd day (H and HMel 500) and even on the 2ed in the case of HMel 50. The obtained results were 87%, 95% and 54% for H, HMel 50 and HMel 500, respectively (Figure 2C).

Only 5 °C temperature significantly blocked germination of maize. Single germinations appeared on the 6-7 days in all seed variants, and by the 14th day of the experiment only 11%, 23%, 25% and 20% of NT, H, HMel 50 and HMel 500 seeds germinated, respectively (Figure 2D). At 5 °C, the marked slowdown in the metabolic activity of seeds was observed, a small number could be considered as germinated, but in the case of every conditioned seed variant it was at least twice more compared to the NT ones. At these chilling conditions hardly any seedling developed. On the basis of the presented germination tests, 5 °C was chosen as a chilling stress temperature for maize seeds and suitable experimental scheme of seed germination and seedling growth conditions was created (see Methods and Materials—C, S, R).

### 2.4. Embryonic Axes Growth

**Figure 3:**
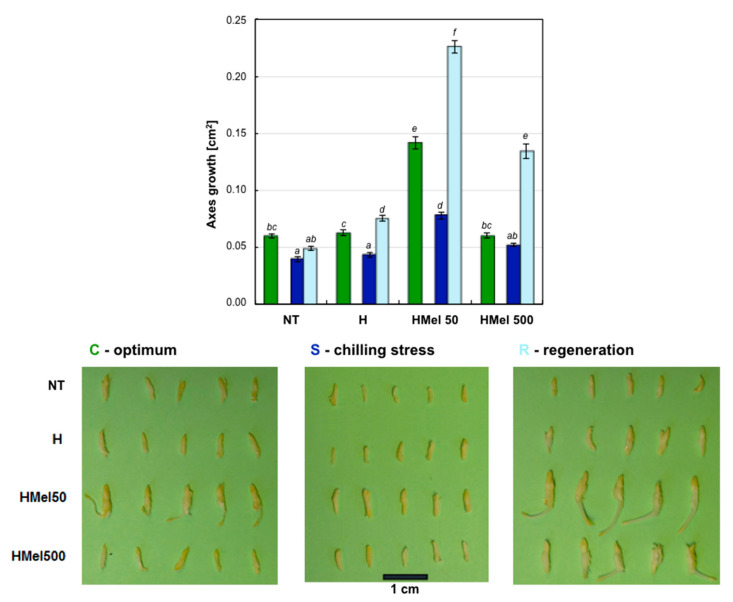
Effect of different maize seed hydropriming treatments on growth of the axes isolated from the non-treated seeds (NT), and those from hydroprimed with water (H) and with MEL water solutions in concentrations: 50 μM (HMel 50) and 500 μM (HMel 500). The seeds were imbibed/germinated in water at 5 °C in darkness for 14 days (S [■]—chilling stress), and subsequently regenerated at 25 °C for 1 day (R [■]—regeneration). Growth was also estimated in axes isolated from the seeds germinated for 24 h under optimal conditions 25 °C (C [■]—optimum, control conditions). The results presented on graph are expressed as means of 20–40 measurements ±SEM. Two-way ANOVA and Duncan’s post-hoc tests were performed. The lowercase letters next to the values show statistical significance at the specified *p*. The results are also presented as a photo panel. GROWTH—ANOVA results: Seed Variant _(NT,H,HMel 50,HMel 500)_ F_(3;348)_ = 368.9, *p* < 0.000001; Conditions _(C,S,R)_ F_(2;348)_ = 267.4, *p* < 0.000001; interaction Seed Variant × Conditions F_(6;348)_ = 67.2, *p* < 0.000001.

During germination at optimal temperature, 25 °C, the embryonic axes of HMel 50 seeds developed the best. In the other variants of seeds (NT, H, Mel 500), their axes growth was similar at 25 °C, but more than half weaker (56–58%) in comparison with HMel 50 (Figure 3).

The chilling stress, 5 °C, significantly inhibited the growth of embryonic axes in NT, H and HMel 500, while in the case of HMel 50 the limiting effect was attenuated and 49, 45 and 33% better results were observed, respectively. Interestingly, the chilled HMel 50 axes were also statistically significantly higher (by 20–23%) compared to the axes of NT, H and HMel 500 seeds developing under optimal conditions of 25 °C. However, when comparing the embryonic axes of HMel 50 seeds growing in optimal conditions with chilled ones, it is clearly visible that in this seed variant the growth was also significantly (45%) limited by cold stress (Figure 3).

Seed conditioning treatments improved their regenerative potential after the chilling stress. Even hydroconditioning with pure water improved growth after the stress removal by 36% compared to the non-conditioned (NT) variant, while in the seeds hydroconditioned with MEL solutions, the effects were spectacular. The embryonic axes in the regenerated HMel 50 and HMel 500 seeds were 4.6 and 2.7 times larger, respectively, compared to the analogous NT ones (Figure 3).

### 2.5. Oxidative Injury Estimation

**Figure 4:**
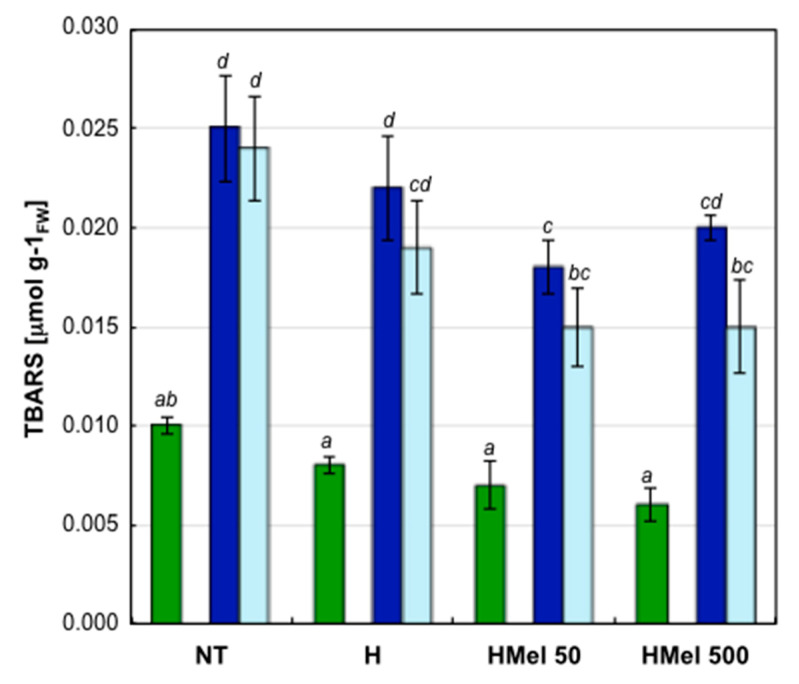
Effect of different maize seed hydropriming treatments on TBARS accumulation in the axes isolated from the non-treated seeds (NT), and those from hydroprimed with water (H) and with melatonin water solutions in concentrations: 50 μM (HMel 50) and 500 μM (HMel 500). The seeds were imbibed/germinated in water at 5 °C in darkness for 14 days (S [■]—chilling stress), and subsequently regenerated at 25 °C for 1 day (R [■]—regeneration). TBARS were also estimated in axes isolated from the seeds germinated for 24 h under optimal conditions 25 °C (C [■]—optimum, control conditions). The results are expressed as means of 6–9 measurements ±SEM. Two-way ANOVA and Duncan’s post-hoc tests were performed. The lowercase letters next to the values show statistical significance at the specified *p*. TBARS—ANOVA results: Seed Variant _(NT,H,HMel 50,HMel 500)_ F_(3;84)_ = 6.57, *p* < 0.000001; Conditions _(C,S,R)_ F_(2;84)_ = 44.07, *p* < 0.000001; interaction Seed Variant × Conditions F_(6;84)_ = 0.80, *p* = 0.57 no statistical significance.

Under optimal germination conditions, the level of compounds formed as a result of lipid peroxidation (TBARS) remained at a low and similar level in embryonic axes from all seed variants (slightly higher in NT) (Figure 4). In the seed embryos incubated for 14 days at chilling 5 °C (S), the degree of lipid oxidation increased significantly 2.7 and 2.5 times in NT and H, respectively, and about 2 times in HMel 50 and HMel 500, comparing with the results obtained under optimal conditions. Although the trend of changes in TBARS level was the same in all seed variants under the given incubation conditions (C, S, R)—so the interaction of Seed Variant × Conditions was not statistically significant—the absolute values always showed statistically significant weaker lipid peroxidation in melatonin pretreated seeds comparing with NT ones, especially under chilling stress (Figure 4). After transferring the germinating seeds from chilling back to the optimal temperature (R), the downward trend of TBARS was observed in all seeds, but in NT this decrease was not statistically significant. After regeneration (R), once again the weakest oxidative damage of lipids was observed in embryonic axes isolated from the seeds pretreated with melatonin (HMel 50 and HMel 500) (Figure 4).

**Figure 5:**
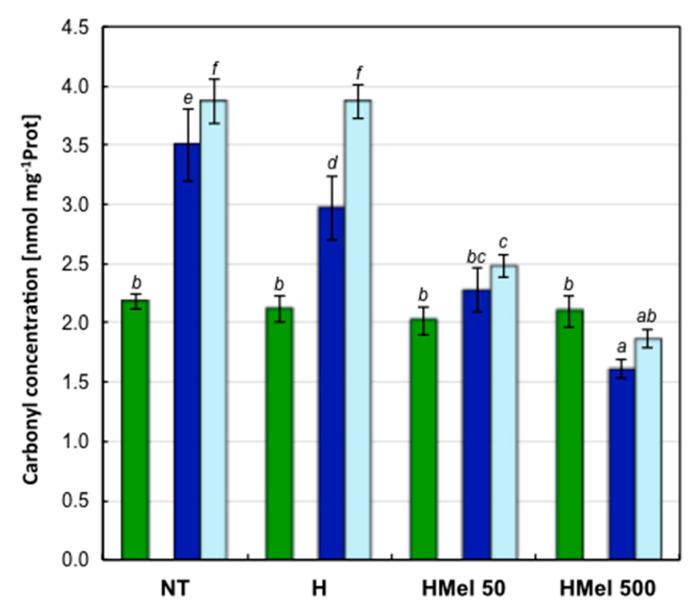
Effect of different maize seed hydropriming treatments on protein oxidation expressed as carbonyl group concentration in the axes isolated from non-treated seeds (NT), and those from hydroprimed with water (H) and with melatonin water solutions in concentrations: 50 μM (HMel 50) and 500 μM (HMel 500). The seeds were imbibed/germinated in water at 5 °C in darkness for 14 days (S [■]—chilling stress), and subsequently regenerated at 25 °C for 1 day (R [■]—regeneration). Proteins and its oxidation were also estimated in axes isolated from the seeds germinated for 24 h under optimal conditions 25 °C (C [■]—optimum, control conditions). The results are expressed as means of 6–9 measurements ±SEM. Two-way ANOVA and Duncan’s post-hoc tests were performed. The lowercase letters next to the values show statistical significance at the specified *p*. Carbonyls—ANOVA results: Seed Variant _(NT,H,HMel 50,HMel 500)_ F_(3;84)_ = 35.9, *p* < 0.000001; Conditions _(C,S,R)_ F_(2;84)_ = 25.0, *p* < 0.000001; interaction Seed Variant × Conditions F_(6;84)_ = 7.83, *p* < 0.000001.

The degree of protein carbonylation in embryonic axes of the seeds germinating under optimal conditions was also similar (Figure 5). The 14-day chilling stress (S) strongly increased the concentration of carbonyl groups in the proteins extracted from NT seed embryos and to a lesser extent from H seed embryos (Figure 5). Unfortunately, the upward trend of this parameter was maintained in the above seed variants even after the stress relief during regeneration (R) (Figure 5). In contrast, in embryos of HMel 50 seeds an insignificant increase in protein carbonylation was observed under chilling stress and after its removal and in the case of HMel 500 even decrease in this parameter (Figure 5). In both cases, the actual reduction of oxidative modifications/damages to proteins probably due to MEL applied to the seeds can be postulated.

### 2.6. Antioxidant and Detoxifying Enzymes Activities

**Figure 6:**
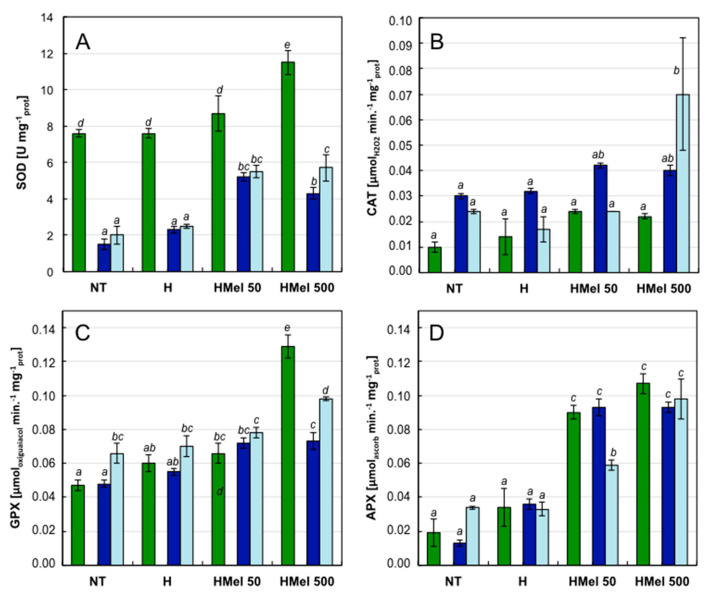
Effect of different maize seed hydropriming treatments on the antioxidant enzymes activities: SOD (**A**), CAT (**B**), GPX (**C**) and APX (**D**) in the axes isolated from non-treated seeds (NT), and those from hydroprimed with water (H) and with melatonin water solutions in concentrations: 50 μM (HMel 50) and 500 μM (HMel 500). The seeds were imbibed/germinated in water at 5 °C in darkness for 14 days (S [■]—chilling stress), and subsequently regenerated at 25 °C for 1 day (R [■]—regeneration). Enzyme activities were also estimated in axes isolated from the seeds germinated for 24 h under optimal conditions 25 °C (C [■]—optimum, control conditions). The results are expressed as means of 6–9 measurements ±SEM. Two-way ANOVA and Duncan’s post-hoc tests were performed. The lowercase letters next to the values show statistical significance at the specified *p*. SOD—ANOVA results: Seed Variant _(NT,H,HMel 50,HMel 500)_ F_(3;79)_ = 39.69, *p* < 0.000001; Conditions _(C,S,R)_ F_(2;79)_ = 167.53, *p* < 0.000001; interaction Seed Variant × Conditions F_(6;79)_ = 2.96 *p* < 0.01. CAT—ANOVA results: Seed Variant _(NT,H,HMel 50,HMel 500)_ F_(3;88)_ = 4.33, *p* < 0 005; Conditions _(C,S,R)_ F_(2;88)_ = 4.32, *p* < 0.01; interaction Seed Variant × Conditions F_(6;88)_ = 2.09, *p* = 0.062 no statistical significance. GPX—ANOVA results: Seed Variant _(NT,H,HMel 50,HMel 500)_ F_(3;82)_ = 75.27, *p* < 0,000001; Conditions _(C,S,R)_ F_(2;82)_ = 16.69, *p* < 0.000001; interaction Seed Variant × Conditions F_(6;82)_ = 14.79, *p* < 0.000001. APX—ANOVA results: Seed Variant _(NT,H,HMel 50,HMel 500)_ F_(3;77)_ = 77.32, *p* < 0.000001; Conditions _(C,S,R)_ F_(2;77)_ = 0.71, *p* = 0.049 no statistical significance; interaction Seed Variant × Conditions F_(6;77)_ = 3.13, *p* < 0.005.

Smaller oxidative damages observed were correlated with a better functioning of the antioxidant enzyme system. Even under optimal conditions, SOD showed higher activity in the HMel 50 and in particular HMel 500 seed embryos (Figure 6A). Chilling significantly reduced SOD activity in the embryonic axes of NT and H seeds and did not increase even after their regeneration at optimal conditions. In the axes of seeds treated with MEL, cold also decreased SOD activity, but only by 1/3 of the control value. Moreover, both in stress and after regeneration, it remained about twice as high as in NT and H seeds (Figure 6A).

There were no significant changes in CAT activity, which was low in embryonic axes isolated from all seed variants. Its activity increased after the chilling stress relief in the case of HMel 500, however, SEM of the obtained values was high, which suggests significant heterogeneity of the material tested with regard to this parameter (Figure 6B).

It seems that in the studied plant material, peroxidases played the main role in eliminating oxidative stress. At optimal conditions 25 °C their activity slightly increased in H and HMel 50 seeds in comparison to NT. The highest activity was observed in HMel 500 ones (Figure 6C). What is important, peroxidases were not chilling sensitive and they kept their activity during the stress. In the non-specific peroxidase test (GPX), in the embryonic axes of NT and H seeds the highest enzymatic activity of all the antioxidant enzymes tested at 5 °C, was observed (Figure 6C). After stress removal during regeneration (R) their activity increased in all seed variants. Moreover, this activity was higher in HMel 50 and HMel 500 as compared to the NT variant. The greatest GPX activity was observed in axes of HMel 500 seeds under optimal conditions: C and R (Figure 6C).

**Figure 7:**
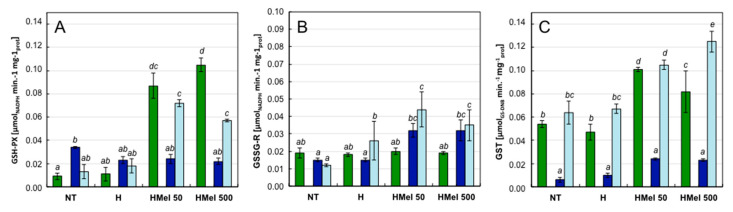
Effect of different corn seed hydropriming treatments on the glutathione-dependent detoxification enzymes activities: GSH-PX (**A**), GSSG-R (**B**), and GST (**C**) in the axes isolated from non-treated seeds (NT), and those from hydroprimed with water (H) and with melatonin water solutions in concentrations: 50 μM (HMel 50) and 500 μM (HMel 500). The seeds were imbibed/germinated in water at 5 °C in darkness for 14 days (S [■]—chilling stress), and subsequently regenerated at 25 °C for 1 day (R [■]—regeneration). Enzyme activities were also estimated in axes isolated from the seeds germinated for 24 h under optimal conditions 25 °C (C [■]—optimum, control conditions). The results are expressed as means of 6–9 measurements ±SEM. Two-way ANOVA and Duncan’s post-hoc tests were performed. The lowercase letters next to the values show statistical significance at the specified *p*. GSH-PX—ANOVA results: Seed Variant _(NT,H,HMel 50,HMel 500)_ F_(3;81)_ = 33.27, *p* < 0.000001; Conditions _(C,S,R)_ F_(2;81)_ = 13.06, *p* < 0.000001; interaction Seed Variant × Conditions F_(6;81)_ = 14.62, *p* < 0.000001. GSSG-R—ANOVA results: Seed Variant _(NT,H,HMel 50,HMel 500)_ F_(3;118)_ = 8.52, *p* < 0.0001; Conditions _(C,S,R)_ F_(2;118)_ = 5.68, *p* < 0.005; interaction Seed Variant × Conditions F_(6;118)_ = 2.60, *p* < 0.01. GST—ANOVA results: Seed _Variant (NT,H,HMel 50,HMel 500)_ F_(3;77)_ = 24.61, *p* < 0.000001; Conditions _(C,S,R)_ F_(3;77)_ = 24.61, *p* < 0.000001; interaction Seed Variant × Conditions F_(6;77)_ = 3.79, *p* < 0.005.

Detailed peroxidase tests showed that MEL applied into the seeds significantly stimulated the APX activity in the axes of HMel 50 and HMel 500 variants under all experimental conditions C, S and R (Figure 6D). In contrast, the activity of APX in NT and H was small, as was the activity of GSH-PX (Figure 7A). Under optimal conditions (C), MEL application significantly stimulated the activity of GSH-PX–10 fold in the case of HMel 50 and 12 fold in the case of HMel 500 comparing with NT. While in the case of GST activity this effect was much less intensive, the increases were 2- and 1.5-fold for HMel 50 and HMel 500, respectively, comparing to NT and H (Figure 7C). Only GSSG activity was similar in all experimental seed variants germinated at optimal conditions (C) (Figure 7B). The studied glutathione-associated enzymes appeared to be sensitive to 5 °C chilling (Figure 7)-the exception was GSSG-R in the HMel 50 and HMel 500 seed axes, where its activity under stress conditions (S) doubled compared to NT and H. High activity of GSSG-R in the melatonin treated seeds was also maintained after stress, during regeneration (R) (Figure 7B). The activity of the detoxifying GST enzyme was also significantly greater in the embryonic axes from melatonin treated seeds (HMel 50 and HMel 500) during recovery after chilling stress (R) (Figure 7C).

### 2.7. Analysis of Cell Distribution in the Determined Cell Cycle Phases

**Figure 8:**
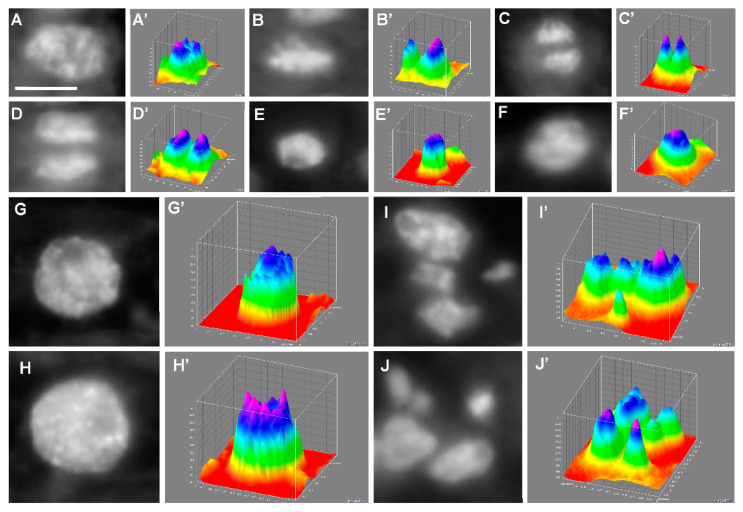
Micrographs (**A**–**J**) and their digital transformations (**A’**–**J’**), of nuclei from *Zea mays* axes basic zone cells fixed in Carnoy’s and stained with DAPI showing: prophase (**A**,**A’**), metaphase (**B**,**B’**), anaphase (**C**,**C’**) and telophase (**D**,**D’**), nucleus during under the G1 (**E**,**E’**) and under the G2 (**F**,**F’**) phases and nuclei in endocycle, i.e., endo S with 6C (**G**,**G’**) and with 8C DNA (**H**,**H’**) as well as nuclei fragmentation (**I**,**I’**), (**J**,**J’**). Scale bar is 10 μm (**A**). The photos and digital transformations represent cells from NT seeds germinated at optimal temperature (Control): (**A**,**A’**), (**B**,**B’**), (**C**,**C’**), (**D**,**D’**) and (**F**,**F’**); from hydroconditioned H seeds germinated at optimal temperature (Control): (**E**,**E’**); from HMel 50 seeds after regeneration phase (R): (**G**,**G’**) and (**H**, **H’**); from hydroconditioned H seeds after regeneration phase (R): (**I**,**I’**) and (**J**,**J’**).

The target of our work was also to determine whether there is a connection between pre-sowing MEL application into maize seeds and endoreplication stimulation in the embryos basic zone. The microcytphotometric measurements of stained DAPI fluorochrome nuclei (Figure 8A–J) and their digital transformation (Figure 8A’–J’) allowed us to observe different karyokinesis phases (Figure 8A–F), endocycle appearance (Figure 8G–H) as well as nuclei fragmentation (Figure 8I–J).

**Figure 9:**
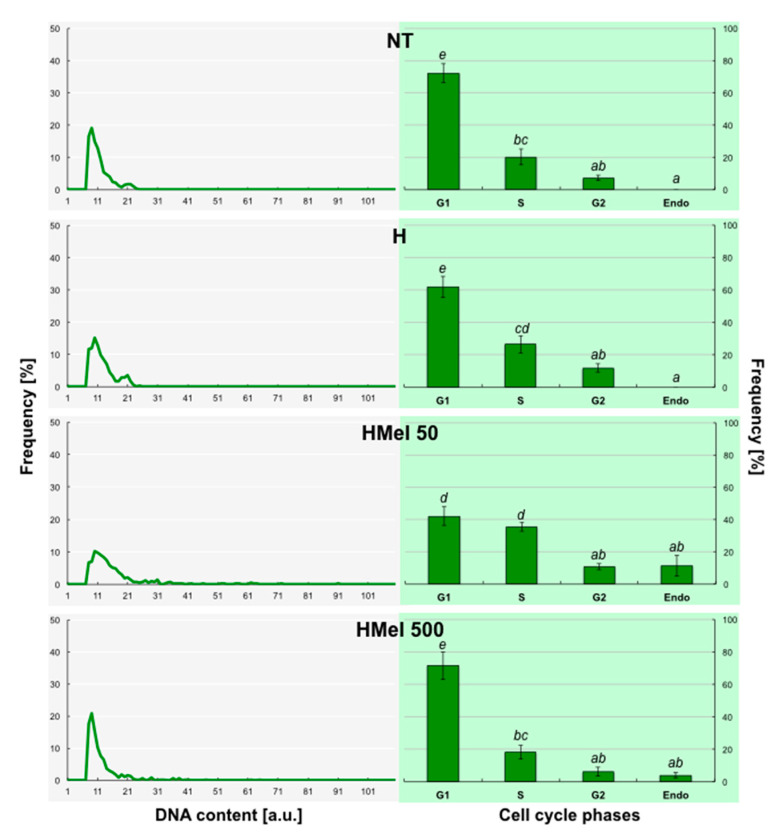
Effect of different maize seed hydropriming treatments on DNA content (line charts) and relative number of cells in G1, G2, S and > 4C phases/Endo (bar graphs) in the axes isolated from non-treated seeds (NT), and those from hydroprimed with water (H) and with melatonin water solutions in concentrations: 50 μM (HMel 50) and 500 μM (HMel 500). The seeds were imbibed/germinated for 24 h under optimal conditions 25 °C (C [■]—optimum, control conditions). The results of cell cycle phase distribution are expressed as means of 5 analyses ±SEM. Two-way ANOVA and Duncan’s post-hoc tests were performed. The lowercase letters next to the values show statistical significance at the specified *p*. Cell cycle phases distribution—ANOVA results: Seed Variant _(NT,H,HMel 50,HMel 500)_ F_(3;64)_ = 0, *p* = 1; Cell Phase _(G1,S,G2,Endo)_ F_(3;64)_ = 136.07, *p* < 0.0000001; interaction Seed Variant × Cell Phase F_(9;64)_ = 4.85, *p* < 0.0001.

The densitometric measurements of DAPI fluorochrome allowed us to conclude that exogenous MEL stimulates endocycle appearance in embryonic axes even during germination at optimal temperature 25 °C (C) (Figure 9). The histograms in Figure 9 are characteristic of undifferentiated tissues, where proliferation takes place and the slow, constant embryo growth ensues. What was important, in the embryos isolated from MEL-treated seeds the polyploid nuclei occurred-especially in HMel 50.

**Figure 10:**
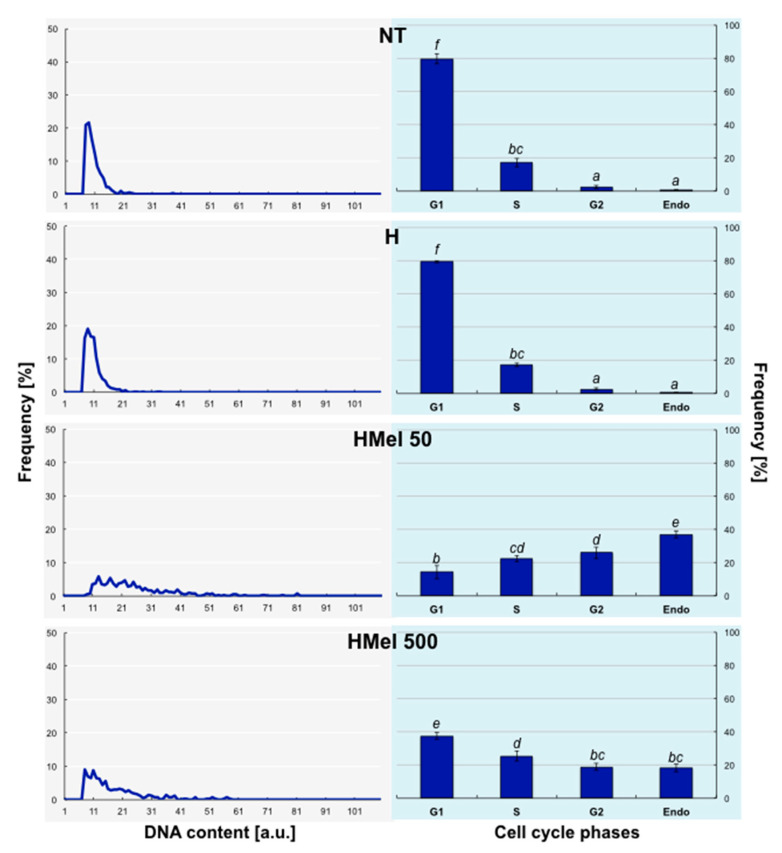
Effect of different maize seed hydropriming treatments on DNA content (line charts) and relative number of cells in G1, G2, S and > 4C phases/Endo (bar graphs) in the axes isolated from non-treated seeds (NT), and those from hydroprimed with water (H) and with melatonin water solutions in concentrations: 50 μM (HMel 50) and 500 μM (HMel 500). The seeds were imbibed/germinated in water at 5 °C in darkness for 14 days (S [■]—chilling stress). The results of cell cycle phase distribution are expressed as means of 5 analyses ±SEM. Two-way ANOVA and Duncan’s post-hoc tests were performed. The small lowercase next to the values show statistical significance at the specified *p*. Cell cycle phases distribution—ANOVA results: Seed Variant _(NT,H,HMel 50,HMel 500)_ F_(3;64)_ = 0, *p* = 1; Cell Phase _(G1,S,G2,Endo)_ F_(3;64)_ = 291.08, *p* < 0.0000001; interaction Seed Variant × Cell Phase F_(9;64)_ = 102.89, *p* < 0.0000001.

The histograms received for seeds germinated at 5 °C (Figure 10) revealed that the proliferation process was disturbed by chilling (S). In NT and H seed variants, cells were mostly stopped in G1 phase, and relatively few cells were observed in other cycle phases. As we supposed, the largest number of endoreplicating cells (about 40% cell nuclei in HMel 50 and 20% in HMel 500) appeared in MEL-treated seeds.

**Figure 11:**
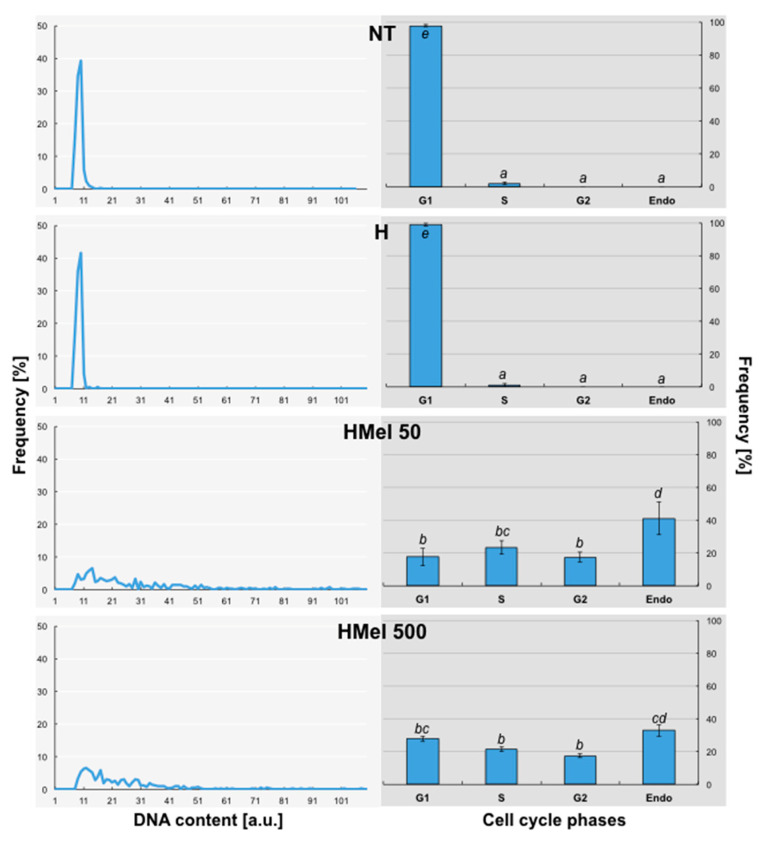
Effect of different maize seed hydropriming treatments on DNA content (line charts) and relative number of cells in G1, G2, S and > 4C phases/Endo (bar graphs) in the axes isolated from non-treated seeds (NT), and those from hydroprimed with water (H) and with melatonin water solutions in concentrations: 50 μM (HMel 50) and 500 μM (HMel 500). The seeds were imbibed/germinated in water at 5 °C in darkness for 14 days and subsequently regenerated at 25 °C for 1 day (R [■]—regeneration). The results of cell cycle phase distribution are expressed as means of 5 analyses ±SEM. Two-way ANOVA and Duncan’s post-hoc tests were performed. The lowercase letters next to the values show statistical significance at the specified *p*. Cell cycle phases distribution—ANOVA results: Seed Variant _(NT,H,HMel 50,HMel 500)_ F_(3;64) =_ 0, *p* = 1; Cell Phase _(G1,S,G2,Endo)_ F_(3;64)_ = 207.15, *p* < 0.0000001; interaction Seed Variant x Cell Phase F_(9;64)_ = 78.56, *p* < 0.0000001.

The measurements made after stress in the recovery period (R) (Figure 11) showed, that in the case of embryonic axes isolated from NT and H seeds, the state observed earlier at chilling stress became prevailing–almost 100% of nuclei were in G1 phase-they did not return to proliferation and nuclei fragmentations were also observed (Figure 8I,J). These seeds were in very bad condition. On the other hand, we detected again a lot of polyploid nuclei–endo-S phase (6C) and tetraploid nuclei (Figure 8G,H, respectively)–in MEL-treated grains, particularly in HMel 50 (about 40%), which resulted in the fast growth and regeneration of seedlings (Figure 3).

## 3. Discussion

Maize (*Zea mays* L.) is a cereal greatly important for people and animals as a nutritional ingredient as well as it may be processed into other industrial biodegradable products [4]. In order to efficient maize cultivation in temperate climate zone, it has to be constantly strengthened by various treatments. We suggest that it is possible with the application of proper methods of pre-sowing seed conditioning.

There are three main types of seeds conditioning: (1) hydroconditioning, (2) osmoconditioning and (3) matriconditioning [8,9,10,13]. All these methods are based on controlled seed hydration leading to limited imbibition, without embryonic axes growth initiation. Final MC has to prevent radical growth and emergence, which allows for seed secondary desiccation to initial MC and then their safety storage until sowing. Final MC, although typical for the seed species, should always be determined experimentally for a given seed lot. 

First, and the simplest priming-water soaking-is a rapid method (time measured in hours), wherein the water availability might be controlled by (i) its quantity given to the seeds (it has to be calculated for known mass of seeds) or (ii) the time of its accessibility (soaking period has to be chosen on the basis of earlier examined kinetics of the seed imbibition—as shown in Figure 1). During osmoconditioning water availability for seeds is limited by a low osmotic potential of the prepared osmotically active solution, which means, that seeds must be incubated longer in it (time measured in days). Similarly, during matriconditioning, low water potential (matrioptential) of specific solid carriers is used. All the mentioned conditioning techniques may be supplemented with an application of growth stimulators, protective components and other bioactive substances (so-call chemical priming).

The seeds with high starch content, including maize (it contains approximately 72% of starch) [4], endures osmopriming badly. Prolonged (measured in days) seed incubation in increased moisture causes losing ability to safe and proper secondary desiccation-so it disturbs the last stage of conditioning. It is probably related to the activation of alpha-amylases and irreversible mobilization of resources by decomposition of starch. As a consequence, unfortunately, osmoconditioned maize seeds germinate worse than the non-conditioned ones (unpublished pre-tests). We documented that experimentally well-matched hydroconditioning technique applied to corn seeds provided satisfying results both in seed germination (Figure 2) and embryonic axes growth (Figure 3). The effects of seed priming were additionally enhanced by using a natural biostimulator–melatonin.

The positive effect of hydropriming, and especially hydropriming with MEL, is clearly visible in germination tests carried out under suboptimal temperature (Figure 2). Additionally, we observed higher regenerative potential of maize embryos after chilling stress 5 °C (Figure 3). The surprising effect of a significant reduction in HMel 500 seed germination under optimal conditions of 25 °C can be explained by excessive reduction of ROS due to a very high concentration of MEL in these seeds (Table 1). It is well known that an excessive amount of ROS generates the oxidative stress, however, ROS at low concentration are also involved in signal transduction for many processes including seed germination initiation [44]. The high concentration of MEL is probably so effective in ROS scavenging at optimal conditions, that too low ROS level in HMel500 seeds at 25 °C disturbs/blocks a phenomenon of the so-called ‘oxidative window’, necessary to sustain the correct signal transduction during early phase of germination [44].

This effect is eliminated at decreasing temperatures (Figure 2B,C), where chilling by increasing ROS in HMel500 cells replenishes the ‘missing’ pool of ROS for proper signal transduction. Probably for this reason HMel 500 seeds germinate better under chilling condition than at the optimal temperature. Only at 5 °C of cold, germination was significantly blocked in all seed variants (Figure 2D).

It must be also noted, that additionally due to the reduced IAA level in HMel 500 seeds (Table 1), their germination was always weaker than in the other (NT, H, HMel 50) seed variants studied (Figure 2).

The level of endogenous melatonin in plants varies depends on the species, organs, age and growth conditions [45,46]. The seeds seem to be exceptionally rich in it. The studied maize grains contained 30–35 ng endogenous MEL per gram fresh weight (Table 1)–that is an average result compared to the richest mustard seeds (129–189 ng g^−1^ [47]). However, such a concentration is incomparably higher than that recorded in animal tissues. As it was found, phytomelatonin is synthesized particularly vigorously in suboptimal conditions and melatonin-rich plant species showed a higher capacity for stress tolerance [48,49,50]. Therefore, it seemed advisable to provide the seeds with this indoleamine preventively. For this purpose, the hydropriming technique was successfully applied (Table 1).

Melatonin is structurally similar to IAA, but the functions of these two molecules differ. MEL more intensely stimulates the growth of aboveground parts of plants [51], although similar to IAA, also provokes root development. All positive effects of MEL activity are especially visible during environmental stresses. In tomato (*Lycopersicon esculentum* Mill.) [52] and brown mustard (*Brassica juncea* L.) [53] treated with exogenous MEL (0.05, 0.01 µM), a slight, transient increase in IAA concentration was observed, but it decreased over time [54]. In contrast, overproduction of MEL in *Arabidopsis thaliana* L. and tomato mutants [55] and treatment of plants with a higher concentration of exogenous MEL (above 0.5 µM) caused a significant decrease of IAA level. The effect of MEL on IAA seems to depend on the MEL quantity used, however, the method of its application and targeted plant organ are also important. In our experiments, much higher concentrations of MEL than in studies discussed above were used. The application of 50 µM MEL to corn seeds did not change the IAA level, only 500 µM MEL reduced its amount to 43% of the control. However, it should be noted, that the hydropriming (seed treatment method) increased the level of IAA in seeds by about 35%, hence the negative correlation was visible only at a higher MEL concentration used. Similar to Wang et al. (2016) [56], we have indirectly demonstrated, that MEL negatively regulates auxin concentration in seeds. However, since various studies show both positive and negative correlation between MEL and IAA, this problem should be considered in a wider network of relationships modulating the expression of auxin-related transcription factors [57,58].

In the presented study, we indicated that MEL applied into the seeds modifies the activity of basic antioxidant and detoxifying enzymes, reducing secondary oxidative stress and chilling injuries.

Cold stress intensifies lipid and protein peroxidation. We have shown that embryonic axes from MEL treated seed variants cumulate significantly less TBARS (products of lipid oxidation) than NT ones, both during stress and after it has subsided (Figure 4). Moreover, the degree of protein carbonylation analysis showed that the seeds treated with MEL had no (HMel 500) or slight (HMel 50) increase in proteins oxidation comparing with its level at optimal temperature condition (Figure 5). Such a protection against chilling stress of protein-lipid membranes and the enzymes functioning in them is certainly the reason for the efficient regeneration and faster growth of the embryonic axes from MEL-treated seeds as documented in Figure 3. The results above are very consistent with those presented by Cao et al. (2019) [59], who conditioned wax maize seeds with MEL (50 and 100 µM) and then observed seedlings grown from them under constant chilling of 13 °C. Moreover, the reduction of ROS accumulation in rice seedlings exposed to 12 °C chilling was also observed after the application of MEL (20 and 100 µM) administered via seed soaking, seedling root immersing or leaves spraying [39]. The positive effects increased with the MEL concentration used. Similarly, under cold stress (2–5 ° C) spraying of wheat [60] and maize [61] seedlings with 1 mM MEL solution, was selected as significant inhibitor of oxidative damage, maintaining membrane functionality. Gao et al. (2018) [38] successfully applied 100 µM MEL over 28 days cold storage of peach fruit. The MEL-treated fruit had a lower level of lipid peroxidation—better membrane integrity than the untreated ones. Thus, the reduction of ROS, lower lipid peroxidation, and decrease in relative electrolyte leakage (a measure of membrane integrity) under the influence of MEL in many plants exposed to various environmental stresses are well documented [56,62,63,64]. It is certainly related to the MEL direct antioxidant properties as well as its influence on the antioxidant enzyme activity.

Our research showed that although SOD from the embryonic axes of maize seeds seems to be sensitive to 5 °C cold, its activity decreased significantly at low temperature and did not regenerate, after the stress subsides, to the activity level achieved at 25 °C by plants incubated under optimal conditions all the time. However, despite that, under and after cold stress, SOD activity was 2–2.5 times higher in pre-sowing treated with MEL seeds than in NT and H ones. (Figure 6A). Especially intensely stimulated by MEL were peroxidases: of ascotrbate (APX—Figure 6D) and of glutathione (GSH-PX—Figure 7A), as well as glutathione S-transferase (GST—Figure 7C); and although the mentioned glutathione-dependent enzymes (GSH-PX and GST) appear to be also sensitive to the low temperature of 5 °C (under cold they performed poorly), thus after transferring the germinating HMel 50 and HMel 500 seeds to 25 °C, they regained efficient functionality very quickly. While glutathione reductase (GSSG-R) in seeds hydroconditioned with MEL was twice as active, both under cold stress and after its disappearance, compared to that in NT and H ones. This helps to maintain a favorable pool of reduced glutathione under stress and during regeneration after its elimination, which is in line with the data published by Bałabusta et al. (2016) [65], that involved cucumber seeds osmoconditioned with MEL. 

Cold is a specific stress—due to deceleration of biochemical reactions and the preservative effect of low temperatures, it is sometimes difficult to observe negative effects when handling it. That is why observations and analyses performed after plants have been transferred to optimal conditions are so important—cold damage is most clearly visible during the regeneration period at a higher temperature.

In a thematically similar work by Cao et al. (2019) [59], a clearly stimulating and directly proportional to the concentration used (MEL 50 and 100 µM), the effect of melatonin applied to wax maize seeds on the activity of all tested antioxidant enzymes, i.e., SOD, CAT, APX and non-specific peroxidases was documented. However, it should be noted, that the chilling stress used in the experiments mentioned above was milder (13 °C) and short-lived (up to 5 days). Probably for this reason it did not strain the activity of cold-sensitive enzymes. Similarly, in the work of Han et al. (2017) [39], MEL 20 and 100 µM solutions, applied via various ways—into the seeds, roots or leaves of rice seedlings, stimulated the activity of SOD, CAT and non-specific peroxidases, especially at the higher concentration of MEL (100 µM) used. However, also in this case, plants were exposed to milder stress of 12 °C for 6 days. Moreover, the examined organs—the leaves of 12- and 14-day-old seedlings—were different than our material (embryonic axes) discussed in this work. Most of the available publications describing the effect of exogenous MEL on the antioxidant enzymes activity concern the developing seedlings of various species, e.g., of wheat [60], corn [61], rice [39]—plant material isolated from germinating seeds was analyzed rarely. The vast majority of works confirm that exogenous MEL applied to plants provokes a positive effect by intensifying (although in slightly various ways) the activity of antioxidant enzymes in face of cold/chilling stress, that in consequence reduces symptoms and damage generated by secondary oxidative stress.

Our latest results [14,42] indicated that MEL seed treatment expediently modified proteome of maize (*Z. mays* L.) embryo during seed germination. The majority of additional proteins were: (i) energy metabolism enzymes, (ii) proteins involved in proteome plasticity via improving protein synthesis, folding, destination and storage, and—most importantly—(iii) defense, anti-stresses, and detoxifying proteins. This explains why seeds hydroconditioned with MEL and seedlings grown from them were stronger in comparison to the non-treated ones, and quickly and efficiently adapted to changing environmental conditions. They were a priori prepared to cope with potential harmful conditions. These results partially explain how melatonin acts in plant stress defense and why various plant species rich in MEL show a higher capacity for stress tolerance [50]. 

Because of not only qualitative, but also a quantitative increase in the protein pool of embryonic axes isolated from the seeds treated with MEL was observed, the questions appeared: how can this happen and especially, how is this possible during chilling stress, when the metabolism is slowing down?

It is well known, that in polyploid cells more efficient and more productive metabolism is observed. Endocycles multiply the number of copies of the desired gene, maximizing mRNA availability and protein production. This phenomenon occurs both during optimal plant development and under harmful environmental conditions [66]. It has been shown that polyploid cells adapt better to environmental changes, including unfavorable ones [67,68]. Polyploidization is associated with the phenomenon of endoreplication—also known as an endocycle. This process is much less understood than the standard cell cycle. However, it has been noticed that plant mutants with suppression of auxin synthesis, transport and signaling show fast mitosis switching to the endocycle and achieve an increased level of polyploidy, which allows cells to grow faster [68]. These known facts, consistent with our results—increased protein synthesis, lower IAA levels, and faster seedling growth due to MEL application into the maize seeds—prompted us to verify whether MEL can also induce endoreplication in growing maize embryonic axes.

Cells that have entered the endocycles, quickly increase their size without losing the materials for building organelles, which would have to be duplicated before classic division. Their enlargement is described with the caryoplasmic factor (the greater the content of genetic material in the nucleus, the greater the cell surface area). This positively influences the rapid growth of plant and the duplication of genes allows the intensification of certain attributes such as flower color, scent, fruit flavor, but also the content of desirable compounds e.g., possessing antioxidant properties [66,67,69].

In the preparations from the basal zone of maize embryonic axes, we observed exceptionally large cells, and after staining with DAPI, the typical endocycle nuclei inside them (Figure 8G,G’,H,H’). They were most often observed in the HMel 50 seed variant and appeared during their incubation under chilling stress (Figure 10) and after it had subsided—during regeneration phase (Figure 11). They were also observed, but less frequently, during the incubation of these seeds in optimal conditions (Figure 9). It should be remembered that at the same moments of the experiment the embryos HMel 50 grew best (Figure 3) compared to the remaining seed variants. Application of MEL at a concentration 10 times higher—500 µM—also provoked the presence of endocycles, but less effectively than in the case of 50 µM MEL seed treatment. It seems that the MEL concentration used is crucial for the occurrence of endocycles, although the sensitivity to it may also vary depending on the organ examined, and certainly the plant species. Wang et al. (2017) [70] showed that high concentration of MEL (1000 µM) suppresses the leaf growth in *Arabidopsis thaliana* L. by the reduction of cell size and cell number. Their further comprehensive analyses suggested that MEL might regulate the leaf growth by inhibiting cell proliferation and endoreduplication too. However, observations mentioned above were carried out under optimal plant growth conditions. Our results are the first that indicate the relationship of MEL application effect with the occurrence of endocycles during, and after chilling stress—as a strategy for increasing plant survival.

It is known, that endoreplication occurs as a natural way for plants to compensate evolutionary losses in small genome and an evolutionary strategy to obtain more of the final products that plants need and synthesize in the different environmental conditions they have to face [71,72,73,74]. Hence it would be useful to have a tool that would allow to induce and control such a strategy to improve the quality and quantity of crops. In our opinion, MEL is a good candidate for this, but further research is necessary in this topic.

## 4. Materials and Methods

### 4.1. Plant Material

Maize seeds (*Zea mays* L. var. Ambrozja) were delivered (TORSEED, Torun, Poland). They were stored in the dark, in dry conditions at room temperature, in tightly closed containers before the experiments started.

### 4.2. Hydropriming

The maize seeds were hydroprimed according to the methods described by Taylor et al. (1998) [43] with our modifications. Seed water content (MC—moisture content) was calculated as the percentage of fresh weight basis (1):

(1) $$\mathrm{MC}=\frac{\mathrm{FW}-\mathrm{DW}}{\mathrm{FW}}\;\cdot \;100\%$$

First, the seeds were weighed (FW) and then after 3 days of their incubation at 108 °C their dry mass (DW) was also weighed. Their initial moisture content was 8.84% ± 0.23.

To establish crucial water content in imbibed seeds before radical protrusion, the seeds were imbibed in different PEG-8000 solutions giving an osmotic potential between −1 and −2 MPa [75]. After 7 days of imbibition with PEG –1.5 MPa the corn seeds did not germinate (there were no radical protrusion observed—the seeds imbibed, but embryos were not grown), so 38.01% ± 1.12 MC was chosen as sufficient for corn seed metabolic activity to occur while preventing radical growth and emergence. 

The technique and hydropriming conditions were determined experimentally as: 3 h of seed soaking at 25 °C (see Results). Oxygenated, distilled water (H) or MEL water solutions 50 (HMel 50) and 500 (HMel 500) µM were used depending on the experimental variant.

Then, the seeds were re-dried at room temperature for the subsequent 3 days (time sufficient for returning to the initial water content) and then used in physiological, biochemical and cytological tests.

### 4.3. Germination Test

The seeds placed on 9-cm diameter Petri dishes with 2 layers of Wht 2 filter paper wetted with distilled water were germinated in darkness at 5–25 °C temperature range. The germination test was performed on samples of 100 seeds: 25 seeds per dish, 4 replicates. A seed was scored as germinated when its coat was broken and a radicle was visible. Germination counts were made daily up to 14 days. The results presented are the means of the values obtained in 4 replicates ± SEM.

### 4.4. Seed Germination and Seedling Growth Conditions

The seeds (variants: NT, H, HMel 50, HMel 500) placed on 9-cm diameter Petri dishes (30 seeds per dish) with two layers of Whatman 2 filter paper (Whatman International Ltd. Maidstone, UK) wetted with distilled water were imbibed/germinated: (i) at optimal temperature 25 °C for 24 h (C—control), (ii) at chilling stress temperature 5 °C for 14 days (S—chilling stress), (iii) at 5 °C for 14 days and then subsequently at 25 °C for 24 h (R, recovery/regeneration after stress). Corn embryonic axes isolated from seeds/young seedlings were used for growth visualization and for biochemical and cytological tests.

The growth of embryonic axes was estimated by analyzing their photographs using ImageJ, an image-processing program designed for scientific multidimensional images, which converts pixels to cm^2^ based on the indexed scale. The results are the means of 20–40 measurements ± SEM.

### 4.5. Melatonin and Indole-3-Acetic Acid Determination

The concentrations of MEL and indole-3-acetic acid (IAA) were determined using high-performance liquid chromatography with electrochemical detection (HPLC-EC).

#### 4.5.1. Extraction Procedure

The seeds (1 g) were homogenized with 5 mL of 50 mM sodium phosphate buffer (pH 8.0) containing 1 mM EDTA. The homogenate was incubated for 30 min. at room temperature in darkness with minimal shaking, in order to ensure complete extraction of indoles. Then, it was centrifuged at 15,000× *g* for 20 min at 8 °C. Initial purification consisted in a two-step solvent-partitioning using ethyl acetate (2 × 10 mL). First at the initial phosphate buffer pH 8.0 and second after pH changing to 3.0. The two organic phases (~10 mL each) were evaporated together under vacuum. The dry residue was re-dissolved in 1 mL of mobile phase, filtered through Supelco ISO-Disc filters (PTEF-4-2.4 mm × 0.2 µm), and frozen at −70 °C until HPLC-EC analysis. When analyzed, 10 μL of each filtrate was injected into the HPLC-EC system.

#### 4.5.2. HPLC-EC Analysis

The HPLC system consisted of a quaternary gradient delivery pump Model 1050 (Agilent Technology, Santa Clara, CA, USA), a sample injector Model 7125 (Rheodyne, Berkeley, CA, USA) and an analytical column ZORBAX SB-C18 3.0 × 250 mm, particle size 5 μm (Agilent Technology) protected by an analytical guard column ZORBAX SBC18 4.6 × 12.5 mm, (particle size 5 μm Agilent Technology). The electrochemical detector model HP 1049 A (Agilent Technology), with glassy carbon working electrode, was used at a voltage setting of +0.80V vs. an Ag/AgCl reference electrode. The detector response was plotted and measured using a Chromstation ver. A.08.03 (Agilent Technology). 

The mobile phase contained 0.15 M sodium dihydrogen phosphate, 0.1 mM EDTA, 0.5 mM sodium octanesulphonic acid, 20% (*v*/*v*) methanol and 5 mM lithium chloride. The mobile phase was adjusted to pH 3.4 with phosphoric acid, filtered through Whatman nylon membrane filter (45 μm) and degassed with helium. The column temperature of 32 °C and flow rate of 0.8 mL min^−1^ were used. 

The concentrations of all compounds in each sample were calculated from the integrated chromatographic peak height on the basis of standards, IAA and MEL, calibration curves, and expressed as ng MEL or μg IAA of g^−1^ fresh weight (FW) of tissues. The results are the means of 6–9 measurements ±SEM.

### 4.6. TBARS Test

Lipid peroxidation was evaluated from the resultant products. Thiobarbituric acid reactive substances (TBARS): aldehydes, mainly malondialdehyde (MDA) and endoperoxides were determined according to the methods described by Hodges et al. (1999) [76]. MDA, routinely used as an indicator of lipid peroxidation, was extracted with 1% (*w*/*v*) trichloroacetic acid (TCA). The reaction with 0.5% (*w*/*v*) thiobarbituric acid (TBA) in 20% (*w*/*v*) TCA was conducted at 95 °C for 30 min. After the samples were chilled in ice, specific absorbance was measured at 532 nm and non-specific absorbance at 600 nm. The results were calculated using a molar absorption coefficient of 155 000 M^−1^ cm^−1^ and expressed as MDA µmol g^−1^ of fresh weight (FW) and they are the means of 9 measurements ± SEM.

### 4.7. OxiProt Test

Protein oxidation was estimated in terms of total carbonyl group content in modified proteins [77]. Protein carbonyl groups reacted with 2,4-dinitrophenyl hydrazine (DNPH) to yield the corresponding 2,4-dinitrophenyl hydrazones, spectrophotometrically measured at 370 nm.

Samples, 250 mg of fresh tissue (axes isolated from germinating seeds), were homogenized in extraction buffer (3 mL) consisting of 100 mM phosphate buffer (pH 7.5), 1 mM EDTA and 2 mM dithiothreitol (DTT) as follows. Each homogenate was centrifuged at 20,000× *g* for 30 min. at 4 °C. Then the supernatant fraction was filtered through Miracloth and used for assays. The protein content was determined according to the Bradford (1976) [78] method using bovine serum albumin (BSA) as a standard. To 0.5 mL of extract (aliquot of 500–800 µg protein), 0.5 mL of 10 mM DNPH in 2 M HCl was added and vortexed every 10 min for 1 h at room temperature. Corresponding protein blanks were prepared by adding 0.5 mL of 2 M HCl instead of DNPH. After incubation, 0.5 mL of 30% (*w*/*v*) TCA was added for protein precipitation, the samples were vortexed, then placed on ice for 15 min. Following centrifugation at 10,000× *g* for 15 min at 4 °C, the supernatant was discarded and the pellets were subjected to extensive washing (three times) with 1 mL of mixture ethanol:ethyl acetate (1:1; *v*/*v*) to remove any unreacted DNPH. Finally, the pellets were solubilized in 1 mL of 6 M guanidine hydrochloride in 5% (*w*/*v*) phosphoric acid at 37 °C (water bath) for 45 min with shaking. 

Carbonyl contents were determined from the absorbance at 370 nm using a molar absorption coefficient of 22,000 M^−1^ cm^−1^ [79]. The results are the means of 9 measurements ± SEM.

### 4.8. Enzyme Extraction Procedure

One gram of fresh weigh of axes was ground in a mortar and homogenized with 0.5 g polyvinylpyrrolidone (PVP) in 5 mL of 0.1 M phosphate buffer (pH 7.5) containing 2.5 mM DTT, 1 mM EDTA, 1.25 mM PEG-4000 and 1 mM phenylmethylsulfonyl fluoride (PMSF). The homogenate was centrifuged at 15,000 rpm for 30 min at 4 °C. The resulting supernatant was filtered through Miracloth, desalted on a PD10 column (Pharmacia, Uppsala, Sweden) and used for the enzyme assays. All steps of the extraction procedure were carried out at 4 °C.

### 4.9. Enzyme Activity Tests

Superoxide dismutase (SOD, EC 1.15.1.1) activity was measured according to Giannopolitis and Ries (1977) [80] with modifications. The reaction mixture contained 2 µM riboflavine, 13 mM methionine, 0.1 mM EDTA, 70 µM nitrotetrazolium blue chloride (NBT) in 0.1 M phosphate buffer (pH 7.5), and 100 µL of the enzyme extract in the final volume of 3 mL. SOD activity was assayed by measuring the ability of the enzyme extract to inhibit the photochemical reduction of NBT. Glass test tubes containing the mixture were illuminated with a fluorescent lamp at 25 °C (Philips MLL 5000 W, Eindoven, the Netherlands). Identical tubes, which were incubated in darkness served as blanks. After illumination for 60 min, absorbance was measured at 560 nm. One unit of SOD was defined as the enzyme activity, which inhibited the photoreduction of NBT to blue formazan by 50 %. SOD activity was expressed as the enzyme unit per milligram of protein [U mgprot^−1^].

Catalase (CAT, EC 1.11.1.6.) activity was measured according to Clairbone (1985) [81] with modifications. The enzyme assay mixture contained 18 mM H_2_O_2_ in 0.1 M phosphate buffer (pH 7.0) and 100 µL of the enzyme extract in the total volume of 2 mL. CAT activity at 25 °C was estimated by the decrease in H_2_O_2_ determined from the absorbance at 240 nm monitored for 100 s. The molar absorption coefficient of 40,000 M^−1^ cm^−1^ [82] was used for calculations. The results were expressed as micromoles of H_2_O_2_ decomposed during 1 min per 1 milligram of protein [µmol min^−1^ mgprot^−1^]. 

Non-specific peroxidases (GPX) activity was measured according to Scebba et al. (2001) [83] with modifications. The reaction mixture contained 2.25 mM guaiacol, 11 mM H_2_O_2_ in 0.1 M phosphate buffer (pH 6.0), and 100 µL of the enzyme extract in the total volume of 2 mL. GPX activity was assayed at 25 °C by following the increase in oxidized guaiacol determined from the absorbance at 470 nm monitored for 300 s. The molar absorption coefficient of 26,600 M^−1^ cm^−1^ [82] was used for calculations. The results were expressed as micromoles of guaiacol oxidized during 1 min per 1 milligram of proteins [µmol min^−1^ mgprot^−1^]. 

Ascorbate peroxidase (APX, EC 1.11.1.11) activity was assayed according to Kato and Shimizu (1987) [82] with modifications. The reaction mixture contained 1.2 mM EDTA, 35 mM H_2_O_2_, 15 mM L-ascorbic acid, 0.1 M phosphate buffer (pH 7.0) and 200 µL of the examined extract. APX activity at 25 °C was estimated by the decrease in ascorbate determined from the absorbance at 290 nm monitored for 300 s. The molar absorption coefficient of 2 800 M^−1^ cm^−1^ [82] was used for calculations. The enzyme activity was expressed as µM L-ascorbic acid decreased in 1 min per 1 mg of proteins [µmol vit C min^−1^ mg prot^−1^]. 

Glutathione peroxidase (GSH-PX, EC 1.11.1.9) activity was determined according to Nagalakshmi and Prasad (2001) [84] with modifications. The assay mixture contained 1 M NaCl, 10 mM EDTA, 10 mM reduced glutathione (GSH), 8 mM NADPH, 25 mM H_2_O_2_, GSSG-R (200 U ml^−1^), 0.1 M phosphate buffer (pH 8.0) and 100 µL of the examined extract. GSH-PX activity at 30 °C was assayed by the decrease in NADPH determined from the absorbance at 340 nm monitored for 10 min. The molar absorption coefficient of 6200 M^−1^ cm^−1^ [85] was used for calculations. The enzyme activity was expressed as µmol of NADPH decreased during 1 min per 1 mg of proteins [µmol NADPH min^−1^ mg prot^−1^].

Glutathione reductase (GSSG-R, EC 1.6.4.2) activity was determined according to Esterbauer and Grill (1978) [86] with modifications. The assay mixture contained 0.5 mM NADPH, 10 mM glutathione disulfide (GSSG), 6.25 mM MgCl_2_ in 0.1 M phosphate buffer (pH 7.5), and 100 µL of the enzyme extract in the total volume of 400 µL. GSSG-R activity at 30 °C was estimated by the decrease in NADPH determined from the absorbance at 340 nm monitored for 20-30 min. The molar absorption coefficient of 6200 M^−1^ cm^−1^ [85] was used for calculations. GSSG-R activity was expressed as micromoles of NADPH decreased during 1 min per 1 milligram of proteins [µmol min^−1^ mgprot^−1^].

Glutathione S-transferase (GST; EC 2.5.1.18) activity was determined according to Habig, et al. (1974) [87] and Nagalakshmi and Prasad (2001) [84] with modifications. The assay mixture contained 10 mM GSH, 100 mM 1-chloro-2,4-dinitrobenzene (CDNB), 0.1 M phosphate buffer (pH 8.0) and 200 µL of the examined extract. Activity of GST was measured at 30 °C by following the increase in GS-DNB at 340 nm during 300 s. The molar absorption coefficient of 9600 M^−1^ cm^−1^ [87] was used for calculations The enzyme activity was expressed as µmol of GS-DNB composed during 1 min per 1 mg of proteins [µmol GS-DNB min^−1^ mg prot^−1^].

SOD, CAT, GPX, APX, GSH-PX, GSSG-R and GST activities of each extract were measured 3 times, and the results presented correspond to the means ± SEM of the values obtained with 3 different extracts (*n* = 9). Protein content of the extracts was determined according to the method of Bradford (1976) [78].

### 4.10. DAPI Staining

The DAPI staining was accomplished according to Byczkowska et al. (2013) [88] and Kaźmierczak (2010) [89] methods. The isolated embryos were fixed in cold Carnoy’s containing 96% ethanol and glacial acetic acid, in proportion 3:1 for 1 h, and then washed with ethanol series of dilutions, finally with distilled water. Then they were cut to slides with cryostat (CM1950, Leica, Buffalo Grove, IL, USA) and stained with 4′,6-diamidine-2′-phenylindole dihydrochloride (DAPI) by 5-min pretreatment with 0.2 M citric acid and 0.1% Tween. The dyeing solution contained 3.2 mM DAPI (2 μg mL^−1^) in mixture of 0.1 M Na2HPO4 and 0.2 M citric acid in 9:1 ratio. After 5 min staining, the axes were washed with the buffer mixture, and subsequently analyzed using a fluorescence microscope (Optiphot-2, Nikon, Melville, NY, USA) with UV2A filter, and photographed using ACT-1 digital camera (Precoptic, Warsaw, Poland). The microphotographs were used for microcytophotometric measurements and digital transformations using ImageJ software.

### 4.11. Statistical Analyses

The results represent the average values ± standard error of the mean (±SEM). The data were analyzed using STATISTICA v.10.0_MR1_PL [StatSoft] software. The two-way or one-way analysis of variance (ANOVA) and then the post-hoc Duncan multiple range test were carried out to find the significant differences which were determined at *p* < 0.01 in each experiment.

## 5. Conclusions

Hydroconditioning is a highly effective technique for application of exogenous MEL into the corn seeds. The level of this indoleamine in pre-sowing treated seeds increases proportionally to the dose of MEL used. However, only a well-chosen dose guarantees success concerning growth and development of young seedling under suboptimal conditions. Unfortunately, MEL overdosing lowers IAA levels in seeds and can disrupt the ROS-dependent signal transduction pathways. 

For maize seed, 50 μM of MEL appears to be the optimal dose. It improves seed germination and embryonic axes growth. That is especially visible during chilling stress (5 °C/14 days) and during regeneration after its discontinuation. 

MEL applied to maize seeds positively modifies the activity of basic antioxidant and detoxifying enzymes (SOD, APX, GSH-PX, GSSG-R and GST). It reduces secondary oxidative stress and injuries generated by them (lipid and protein oxidation). 

It was shown for the first time that MEL induced another/novel strategy to overcome chilling stress—it was polyploidization induced by endoreplication, observed in the cells of basal zone in maize embryonic axes. Polyploidy cells adapt much better to environmental changes, including different unfavorable ones. 

To sum up, it seems that MEL can be an inexpensive, safe and easy-to-apply plant biostimulator that could be widely used in organic farming. 

## Figures and Tables

**Table 1:** Effect of different seed hydropriming treatments on melatonin and IAA content in maize seeds. Seeds were hydroconditioned with water (H) or with melatonin water solution at concentrations: 50 and 500 µM (HMel 50 and HMel 500 respectively). Control seeds were not primed (NT). The results were expressed as means of 9–12 measurements ±SEM. One-way ANOVA and Duncan’s post-hoc test were performed. The lowercase letters next to the values show the statistical significance at the specified *p*.

Seed Variants	Melatonin [ng gFW^−1^]	IAA [µg gFW^−1^]
NT	35 ± 5 *a*	2.63 ± 0.21 *b*
H	31 ± 2 *a*	3.50 ± 0.4 *c*
HMel 50	1058 ± 58 *b*	2.07 ± 0.13 *b*
HMel 500	20,830 ± 902 *c*	1.13 ± 0.14 *a*

Melatonin—ANOVA results: Seed Variant _(NT,H,HMel 50,HMel 500)_ F_(3;25)_ = 675.0, *p* < 0.000001. IAA—ANOVA results: Seed Variant _(NT,H,HMel 50,HMel 500)_ F_(3;34)_ = 13.96, *p* < 0.000005.

## Abbreviations

^1^O_2_*	singlet oxygen
ANOVA	statistical analysis of variance
APX	ascorbate peroxidase
BSA	bovine serum albumin
C	optimal, control conditions
CAT	catalase
CDNB	1-chloro-2,4-dinitrobenzene, citric acid
DAPI	diamidine-2′-phenylindole dihydrochloride
DTT	dithiothreitol
DNA	deoxyribonucleic acid
DNPH	2,4-dinitrophenyl hydrazine
DW	dry weight
EDTA	ethylenediaminetetraacetic acid disodium salt
FW	fresh weight
GSH	reduced glutathione
GSH-PX	glutathione peroxidase
GSSG	glutathione disulfide
GSSG-R	glutathione reductase
GST	glutathione S-transferase
H	hydroconditioning with water
H_2_O_2_	hydrogen peroxide
HCl	hydrochloric acid
HMel 50	hydroconditioning with MEL solutions at 50 μM
HMel 500	hydroconditioning MEL solutions at 500 μM
HPLC-EC	High Pressure Liquid Chromatography with Electrochemical Detection
IAA	indole-3-acetic acid
MC	moisture content
MDA	malondialdehyde
MEL	melatonin
NBT	nitrotetrazolium blue chloride
NT	non-treated, control seeds
NADPH	β-nicotinamide adenine dinucleotide phosphate reduced form
NBT	nitrotetrazolium blue chloride
NO	nitric oxide
OH*	hydroxyl radical
ONOO^–^	peroxynitrite anion
PEG	polyethylene glycol
PMSF	phenylmethylsulfonyl fluoride
PVP	polyvinylpyrrolidone,
R	regeneration after stress
RNS	reactive nitrogen species
ROS	reactive oxygen species
S	chilling stress
SEM	standard error of the mean
SOD	superoxide dismutase
TBA	thiobarbituric acid
TBARS	thiobarbituric acid reactive substances
TCA	trichloroacetic acid and tween
